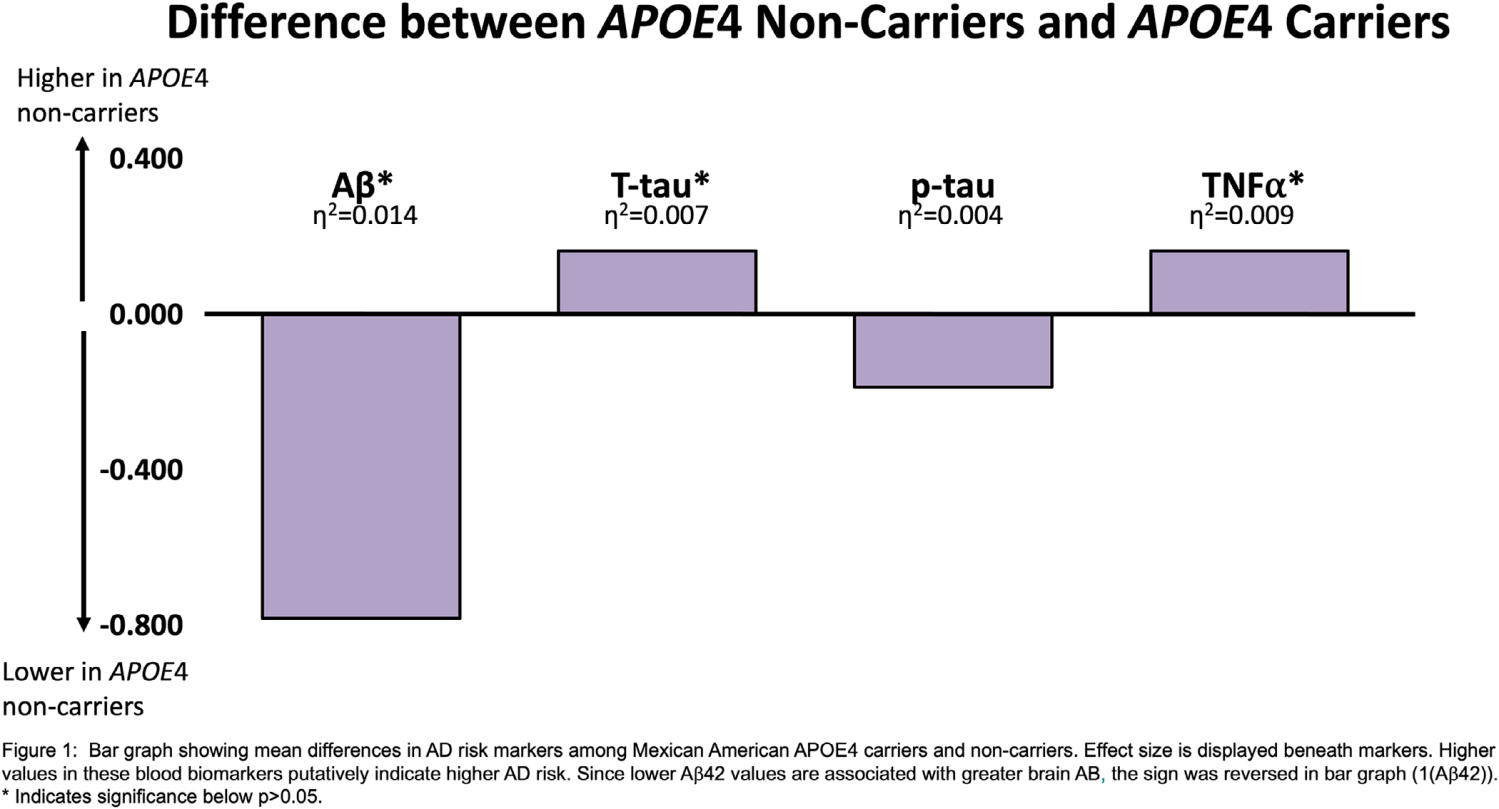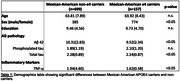# Characterizing the Effects of APOE4 on AD Risk Markers in Mexican‐Americans

**DOI:** 10.1002/alz.091890

**Published:** 2025-01-09

**Authors:** Joey A Contreras, Vahan Aslanyan, Kimberly Espejo, Judy Pa

**Affiliations:** ^1^ Alzheimer's Disease Cooperative Study (ADCS), University of California San Diego, La jolla, CA USA; ^2^ Department of Population and Public Health Sciences, Keck School of Medicine, University of Southern California, Los Angeles, CA USA; ^3^ Alzheimer's Disease Cooperative Study (ADCS), University of California San Diego, San Diego, CA USA; ^4^ Alzheimer's Disease Cooperative Study (ADCS), University of California, San Diego, La Jolla, CA USA

## Abstract

**Background:**

There is a growing need to determine if APOE status infers the same risk for Alzheimer’s disease (AD) in a Hispanic/Latino cohort as it would for a White cohort. To address this, we must first establish the relationship between APOE4 and known at‐risk markers for AD, such as amyloid beta (Aβ), tau, and inflammation. The following analysis compares these markers in non‐e4 carriers and e4 carriers within a Hispanic/Latino Cohort to help answer the question of whether APOE status correlates to AD pathology and inflammation in the same way as we have seen in other populations.

**Method:**

Using baseline data from the Health and Aging Brain Study‐Health Disparities (HABS‐HD), a one‐way ANCOVA was conducted to determine group differences between Ab42, total tau, phosphorylated tau, and inflammatory marker Tumor Necrosis Factor (TNF)‐a among Mexican American APOE4 carriers and non‐carriers while controlling for sex and education. Outlier analysis was also conducted for inflammatory marker, TNF‐alpha. Participants with missing data and TNF‐α outlier values were excluded.

**Result:**

The data used included 856 Hispanic participants, 699 APOE4 carriers (65%female, mean age ±SD=63.81(7.89)), and 157 non‐carriers (68% female, mean age ±SD=63.92(8.43)). There was significant differences between Mexican American APOE4 carriers and non‐carriers among AD pathology markers; Ab42(p=0.002), total tau(p=0.037), and inflammatory marker, TNF‐α(0.003) (Table 1). The bar graph illustrates mean differences in risk markers evaluated between both groups (Figure 1).

**Conclusion:**

The obtained results indicate that individuals without the APOE4 gene variant exhibit elevated levels of total tau and increased inflammation (as indicated by TNF‐a), contradicting findings from prior literature. This suggests that genetic risk factors may not be as ubiquitous as previously reported and that the extent of the risk associated with the e4 variant could be influenced by race/ethnicity. Subsequent studies will contribute to this discovery by augmenting the sample size within the existing dataset and directly comparing it to a white cohort possessing more comprehensive neuroinflammatory biomarker data. Further details about the HABS‐HD cohort are provided in the description below.